# The role of the fractional flow reserve in the coronary steal phenomenon evaluation caused by the coronary-pulmonary fistulas: case report and review of the literature

**DOI:** 10.1186/s13019-020-1073-x

**Published:** 2020-02-03

**Authors:** Zhiwei Huang, Zhihong Liu, Shaodong Ye

**Affiliations:** Fuwai Hospital, National Center for Cardiovascular Diseases, Chinese Academy of Medical Sciences and Peking Union Medical College, No. 167 Beilishi Road, Xicheng District, Beijing, 100037 China

**Keywords:** Coronary-pulmonary artery fistulas, Coronary arteriography, Fractional flow reserve

## Abstract

**Background:**

Congenital coronary-pulmonary fistulas (CPFs) are commonly unilateral; however, bilateral and multilateral fistulas are relatively rare. The steal phenomenon aroused from bilateral or multilateral CPFs, and was uncertain and seldom reported. We possess a new tool to assess the hemodynamic significance of coronary artery fistulas. This study aimed to describe the clinical presentation, diagnostic modalities, and management of the coincidentally detected congenital bilateral CPFs.

**Case presentation:**

A case of a 52 year-old female with 10 years history of typical palpitations and chest tightness was presented. The selective coronary arteriography showed a right dominant coronary circulation without significant stenosis; however, with anomalous vessels originating from the proximal right and left anterior descending coronary arteries, draining into the pulmonary artery through a plexus of small vessels. We introduced the fractional flow reserve (FFR) to evaluate the hemodynamic significance of CPFs. The patient was successfully treated with coil embolization.

**Conclusions:**

We presented the case of a female with typical palpitations and chest tightness due to the steal phenomenon that aroused from bilateral CPFs. The fistulas were safely and successfully closed by coil embolization. We showed a new tool for the sophisticated evaluation of the hemodynamic significance of CPFs using FFR measurement and temporary occlusion of the fistula with a standard balloon. FFR could be a promising means for the treatment of decision making of the CPFs.

## Background

The coronary-pulmonary artery fistulas (CPFs) are links between one or more of the coronary and pulmonary arteries, and they are rare and usually arise from the left anterior descending or the right coronary artery. The CPFs are mostly asymptomatic for a long time, and mainly if they are small, the frequency of the symptoms and particularly the complications rise with age. The clinical presentation could include dyspnea, angina, endocarditis, arrhythmias, heart failure, myocardial ischemia, thrombosis or myocardial infarction [1]. Previously, the treatment of CPFs patients depends on the size, anatomic features of the fistula, and the presence of symptoms. For a long time, it lacks an effective tool to evaluate the hemodynamic significance of CPFs. In the present study, the clinical data of one patient with CPFs, as confirmed by the selective coronary angiography at our hospital, are reported and analyzed in order to raise awareness and improve the CPF diagnosis. Further, we introduce a new approach called the fractional flow reserve (FFR), which is widely used in the critical coronary lesions for the assessment of the hemodynamic significance of CPFs.

## Case presentation

**Fig. 1:**
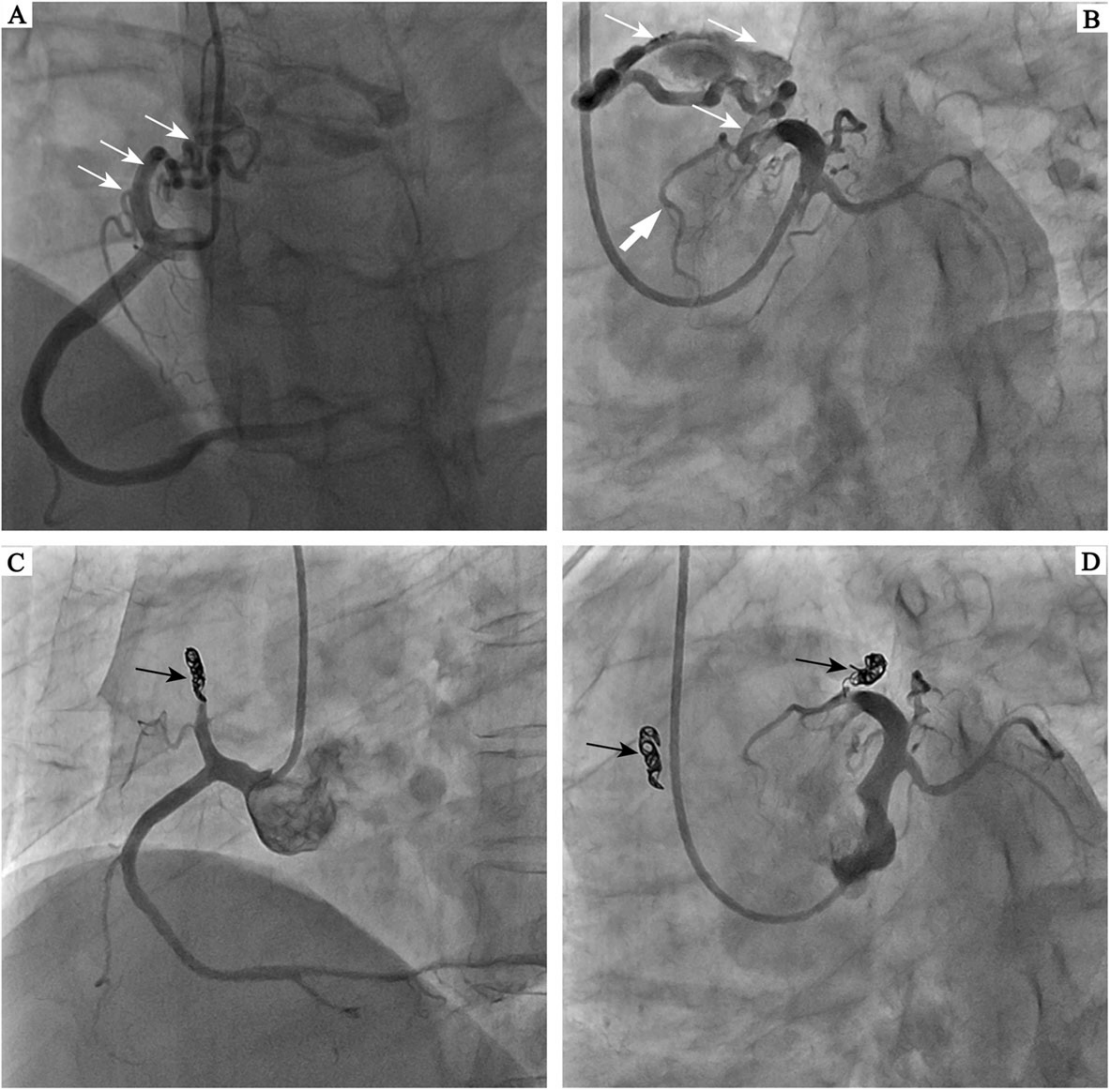
**a** Coronary angiography demonstrating a coronary artery fistula between the right coronary artery (RCA) and the pulmonary artery (PA) by white arrows. **b** Coronary angiography demonstrating a coronary artery fistula between the left anterior descending artery (LAD) and the main pulmonary artery by small white arrows. Moreover, coronary angiography demonstrating a poor development of the distal LAD by bold white arrows. **c** Shunt between the RCA and PA is blocked by coil embolization (black arrow). **d** Shunt between the LAD and PA is blocked by coil embolization (black arrow)

**Fig. 2:**
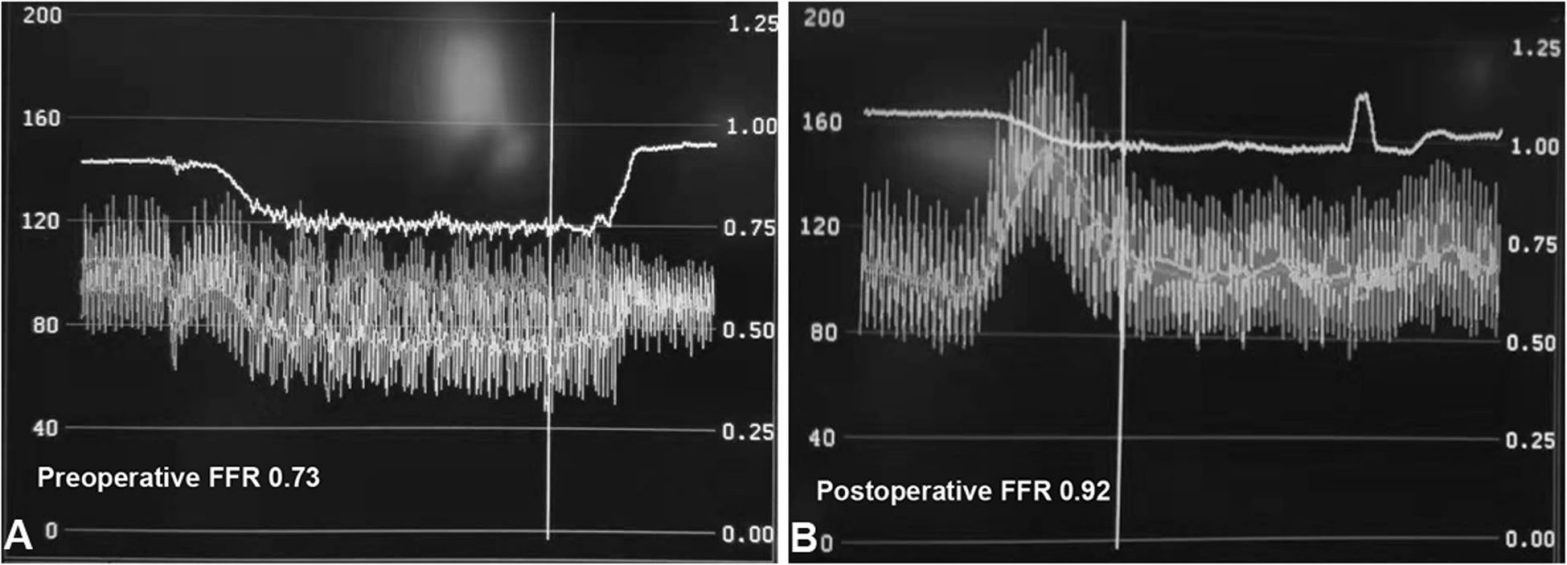
**a.** The FFR of preoperaton in the LAD. **b.** The FFR of postoperaton in the LAD

A 52 year-old female with hyperlipidemia history was admitted to our hospital with 10 years history of typical palpitations and chest tightness. Formerly, she was diagnosed with coronary artery disease (CAD) and received its therapeutic regimen. But her symptoms did not relieve, so the patient was transferred to our hospital for the diagnosis confirmation as well as to provide effective treatment. Moreover, the physical examination showed no abnormal findings, and the cardiac auscultation revealed normal first and second heart sounds, with murmurs not noted. The electrocardiogram (ECG) showed nonspecific ST-T wave flattening and the chest x-ray showed the absence of heart dilation with normal pulmonary vascularity. The transthoracic echocardiogram indicated that the cardiac structure and function were normal without abnormal shunt. Although the exercise testing result was negative, the subject asserted on the performance of a selective coronary angiography. The selective coronary arteriography (Fig. 1a) presented a right dominant coronary circulation without significant stenosis; however, with anomalous vessels originating from the proximal right and left anterior descending coronary arteries, draining into the pulmonary artery through a plexus of small vessels. Similarly, the selective coronary arteriography showed the proximal part of the left anterior descending coronary expansion, and the dysplasia of the distal left anterior descending coronary artery (Fig. 1b). The pulmonary-systemic flow ratio (Qp/Qs) was 1.36 by right heart catheterization (RHC). The FFR in the distal left anterior descending (LAD) was 0.73 under maximal hyperemia, increasing to 0.92 while under temporary experimental occlusion of the fistula using a 3.0/15 mm standard balloon catheter (Fig. 2). However, the FFR in the right coronary artery (RCA) manifested no significant change. Subsequently, we told the patient that a coil embolization of the fistula in the LAD should be performed. To our surprise, the subject insisted on closing both the CPFs. After familial discussion and receiving the patient’s informed consent, the coil embolization of the fistula was performed in the procedure, using the 2 Fibered Platinum Coil™ embolization coils (Boston Scientific, USA) 4 mm–15 cm in the fistula of the right coronary artery to the pulmonary artery and the 3 Fibered Platinum Coil™ embolization coils (Boston Scientific, USA) 2 mm × 5 mm × 5.8 mm in the fistula of the left anterior descending coronary artery to the pulmonary artery (Fig. 1c and d). After the procedure, the patient felt that her symptoms were relieved and reported absence of any discomforts during the 1-year follow-up period.

## Discussion

The first reported case of a CAF was in the year 1865 by Krause [2]. The coronary artery fistula is a rare coronary artery anomaly. The overall population prevalence of the coronary artery fistula is unclear, and it occurs as an incidental finding in 0.13–0.18% of the coronary artery angiography [3, 4]. The bilateral CPFs were previously described [5]; nevertheless, multiple CPFs arising from all 3 major coronary arteries draining into the pulmonary artery were extremely rare. In our study, we reported about a female with bilateral CPFs and what strategy we had undertook.

As for the experienced sonologist, a transthoracic echocardiography may find continuous turbulent flow in the pulmonary artery during the diastolic phase in the short axis view. Small or moderate CPFs are often missed by most sonologists. Furthermore, it is difficult to find the origin of the turbulent color flow. Generally, the CPFs are detected by the multidetector computed tomography (MDCT) and coronary angiography. MDCT is a noninvasive imaging technique that has been successfully utilized for visualization of the unilateral and multilateral fistulas and for the diagnosis of coronary artery anomalies [6, 7]. Coronary angiography remains the gold standard method for CPFs, and it can be used to evaluate the size, number of fistulas, and anatomical features of the fistulous tract [8].

The results of the CPFs’ origin were inconsistent. The right coronary artery, or its branches, was the site of the fistula in 50–55% of cases. Additionally, the left coronary artery was involved in about 35–40%, and both the coronaries were involved in 5–10% [9]. However, some studies reported that most of them originated from the proximal LAD [10, 11]. The CPFs seldom cause symptoms in the first 2 decades of life, but after that, the symptoms and complications may likely increase, including dyspnea, angina, endocarditis, arrhythmias, high output cardiac failure, myocardial ischemia, thrombosis or myocardial infarction.

In our case, palpitations and chest tightness were the chief complaints of the patient. We speculated that these symptoms may be associated with increased pulmonary blood flow and dysplasia of distal LAD. The steal phenomenon arousing from the CPFs was controversial. The coronary steal theory is that runoff from a comparatively large proximal arterial segment occurs preferentially through a lower resistance vascular bed (like a fistula), which reduces the flow to the higher resistance nutrient coronary branches. The hemodynamic balance between the fistulous runoff and the nutrient branches improves when the cardiac load is increased (which causes coronary arteriolar vasodilation), to the extent that effort related angina or ischemia is usually absent. In addition, if functional ischemia or infarction does occur in a patient with a coronary fistula, this is generally the result either of coronary occlusive disease in the nutrient branches or of fistulous tract degeneration [12]. However, the previous view was challenged. Some studies supported that the CPFs cause cause myocardial ischemia [13] an ipsilateral myocardial infarction in the absence of an obstructive CAD due to the coronary steal phenomenon [14] or association with a thrombotic CAD [15]. Despite a debate about the occurrence of the continuous steal phenomenon in the CPFs, FFR remains a promising diagnostic technique that has recently been reported to successfully depict the coronary steal phenomenon. In the CPFs, despite the presence of normal pulmonary artery pressure and small magnitude left-to-right shunt, a myocardial infarction can still develop without the stenotic CAD [14]. Accurate functional evaluation of the CPFs using the FFR measurement under maximal hyperemia of the distal segment of the nutrient coronary artery during temporary balloon occlusion of the fistulous vessel demonstrated the steal phenomenon.

In the past, the treatment of CPF patients depends on the size and anatomic features of the fistula, presence of symptoms, patient’s age, and presence of other cardiovascular diseases. Symptomatic fistula should be occluded by percutaneous intervention or by surgical ligation. Further, occlusion is reasonable for the management of patients with moderate or large coronary artery fistulae without clinical symptoms [16]. Nevertheless, the best approach to the asymptomatic CPFs remains controversial. Nonetheless, at present, we possess a new tool to assess the hemodynamic significance of coronary artery fistulas.

## Conclusions

In conclusion, we presented the case of a female with typical palpitations and chest tightness due to coronary fistula from the proximal LAD and RCA to the pulmonary trunk. The fistulas were safely and successfully closed by coil embolization. In addition, we presented a new tool for sophisticated evaluation of the hemodynamic significance of the CPFs using the FFR measurement and temporary occlusion of the fistula with a standard balloon. The FFR could be a promising means for the treatment of decision making of the CPFs.

## Data Availability

The datasets used and/or analyzed during the current study are available from the corresponding author on reasonable request.

## Abbreviations

CAD: Coronary artery disease
CPFs: Congenital coronary-pulmonary fistulas
ECG: Electrocardiogram
FFR: Fractional flow reserve
LAD: Left anterior descending
MDCT: Multidetector computed tomography
RCA: Right coronary artery
